# Monozygotic twins discordant for homologous Robertsonian translocation trisomy 21 of 46, XX, + 21, der (21;21) (q10; q10) in a twin-to-twin transfusion syndrome, case report

**DOI:** 10.1186/s12884-021-03587-x

**Published:** 2021-01-30

**Authors:** Dingya Cao, Jimei Sun, Nan Li, Zhihua Li, Weiqiang Liu, Min Chen

**Affiliations:** Department of Obstetrics and Gynecology, Department of Fetal Medicine and Prenatal Diagnosis, Key Laboratory for Major Obstetric Diseases of Guangdong Province, The Third Affiliated Hospital of Guangzhou Medical University, 63 Duobao Road, Liwan District, Guangzhou, 510150 China

**Keywords:** Monozygotic twins, Monochorionic diamniotic, Homologous Robertsonian translocation, Trisomy 21, Discordant karyotype, Discordant anomaly, Twin-to-twin transfusion syndrome

## Abstract

**Background:**

Monozygotic twins are nearly identical in genotype and phenotype because monozygotic twins arise from one fertilized oocyte. In all cases of discordant karyotype in monozygotic twins, trisomy 21 accounts for about one in 385,000. Monozygotic twins discordant for Robertsonian translocation trisomy 21 of the der (21;21)(q10;q10), in which the additional chromosome originates from the father is rare.

**Case presentation:**

A 28-year-old parous woman, G3P1A0, came to our institution for a dating scan at 8 weeks of gestation. The transvaginal ultrasound examination demonstrated a monochorionic diamniotic pregnancy. She and her husband were healthy, with no family history of trisomy 21 or other congenital diseases. The ultrasound examination of nuchal translucency thickness was discordant in twins at 13 weeks (twin A, NT 1.4 mm with CRL being 65 mm; twin B, NT 7.8 mm with CRL being 69 mm). At 17^+ 4^ weeks, twin A was normal, but ventricular septal defect and the hypoplastic left heart was detected in twin B. The deepest vertical pocket was 18 mm in twin A (oligohydramnios) and 102 mm in Twin B (polyhydramnios). The bladder in twin A was absent. Ultrasound findings indicated TTTS Stage II. Amniocentesis was performed for the two fetuses. The karyotyping results revealed 46, XX in twin A but 46,XX,+ 21,der (21;21)(q10;q10) in twin B.

For twin B, the parents opted for selective fetal termination by radiofrequency ablation. The procedure was uneventful. At 40^+ 5^ weeks, twin A was born with a birth weight of 4120 g by vaginal delivery.

**Conclusions:**

The early detection of discordant karyotype and twin-to-twin transfusion syndrome is beneficial to the early intervention. In monozygotic twins with a discordant anomaly, the discordant karyotype should be considered.

**Supplementary Information:**

The online version contains supplementary material available at 10.1186/s12884-021-03587-x.

## Background

Monochorionic-diamniotic (MCDA) twins are concordant in karyotypes and phenotype because they developed from a single zygote. The discordance for aneuploidy in monozygotic twins is rare.

Trisomy 21 (Down Syndrome, DS) is the most common chromosomal abnormality. Individuals with DS are commonly characterized by unique neurocognitive and neurobehavioral profiles that emerge within specific stages in the developmental continuum [1]. The incidence of DS observed in twin pregnancies is lower than expected [2]. In the three different types of cytogenetic abnormalities, only 2–4% cases are Robertsonian Translocation Trisomy 21 [3].

We reported a MCDA twin pregnancy complicated with Twin-to-Twin Transfusion Syndrome (TTTS) and discordant for 46, XX,+ 21, der (21;21)(q10;q10). For the best of our knowledge, there is no such case reported in the literature.

## Case presentation

A 28-year-old woman, gravida 3 para 1 abortus 0, came to our unit for a dating scan. She and her husband were healthy and had no family history of DS or other congenital diseases. Her first child was a healthy boy; however, the second pregnancy ended in spontaneous miscarriage around the 5th week.

In this third spontaneous pregnancy. The transvaginal ultrasound examination at 8 weeks of gestation revealed a monochorionic-diamniotic pregnancy. The nuchal translucency (NT) thickness of twin A was normal (1.4 mm) with crown-lump length (CRL) being 65 mm, while a markedly increased NT (7.8 mm) was detected in twin B (CRL 69 mm) at 13 weeks’ NT scan (Supplementary Fig. 1). Since high NT thickness in the monozygotic twin pregnancy was at increased risk for Trisomy 21, other chromosomal abnormalities, TTTS, etc. We recommended follow-up scan every 2 weeks [4].

At 17^+ 4^ weeks, multiple abnormalities: ventricular septal defect (VSD) and the hypoplastic left heart was identified in twin B (Supplementary Fig. 2). The Doppler examination of the tricuspid valves and ductus venous was normal. The morphology of twin A was unremarkable. Estimated fetal weight was 183 g for Twin A, 163 g for Twin B, respectively. The weight discordance was 10.9%. We could identify the twins’ bladder with ultrasound, but the twins showed an unbalanced deepest vertical pocket (DVP) (26 mm in twin A and 89 mm in twin B). Amniocentesis from both sacs was performed. At 18^+ 4^ weeks, a detailed morphology scan showed that the bladder was not visualized in twin A and VSD was apparent in twin B. The DVP was 18 mm in twin A and 102 mm in twin B. A diagnosis of TTTS of stage II was made [4].

The multiplex ligation-dependent probe amplification (MLPA) study revealed normal karyotype in twin A but trisomy 21 in twin B. Further molecular genetic analysis was done to confirm the twins’ monozygosity with 13 short tandem repeat (STR) markers: D3S1358, D5S818, D13S317, D16S539, D2S1338, D6S1043, D19S433, D12S391, D18S51, D8S1179, D21S11, D7S820, and CSF1PO (Table 1). Polymorphic microsatellite markers were well-informative and identical between twins, indicative of monozygosity.

**Table 1:** Results of STR analysis

Markers	Alleles (father/twinA/twinB/ mother)	Location
D3S1358	16,17/15,16/15,16/15,15	p21.31
D5S818	11,13/11,12/11,12/11,12	q23.2
D13S317	8,12/8,12/8,12/8,8	q31.1
D16S539	11,13/9,13/9,13/9,10	q24.1
D2S1338	23,24/23,23/23,23/23,24	q35
D6S1043	14,18/17,18/17,18/11,17	q15
D19S433	13,14.2/13,14/13,14/14,15.2	q12
D12S391	21,23/19,23/19,23/18,19	p13.2
D18S51	15,16/15,16/15,16/15,18	q21.33
D8S1179	10,10/10,16/10,16/16,16	q24.13
D21S11	30,32/29,32/29,32/29,29	q21.1
D7S820	8,10/10,10/10,10/10,12	q21.11
CSF1PO	12,12/12,12/12,12/11,12	q32

**Fig. 1:**
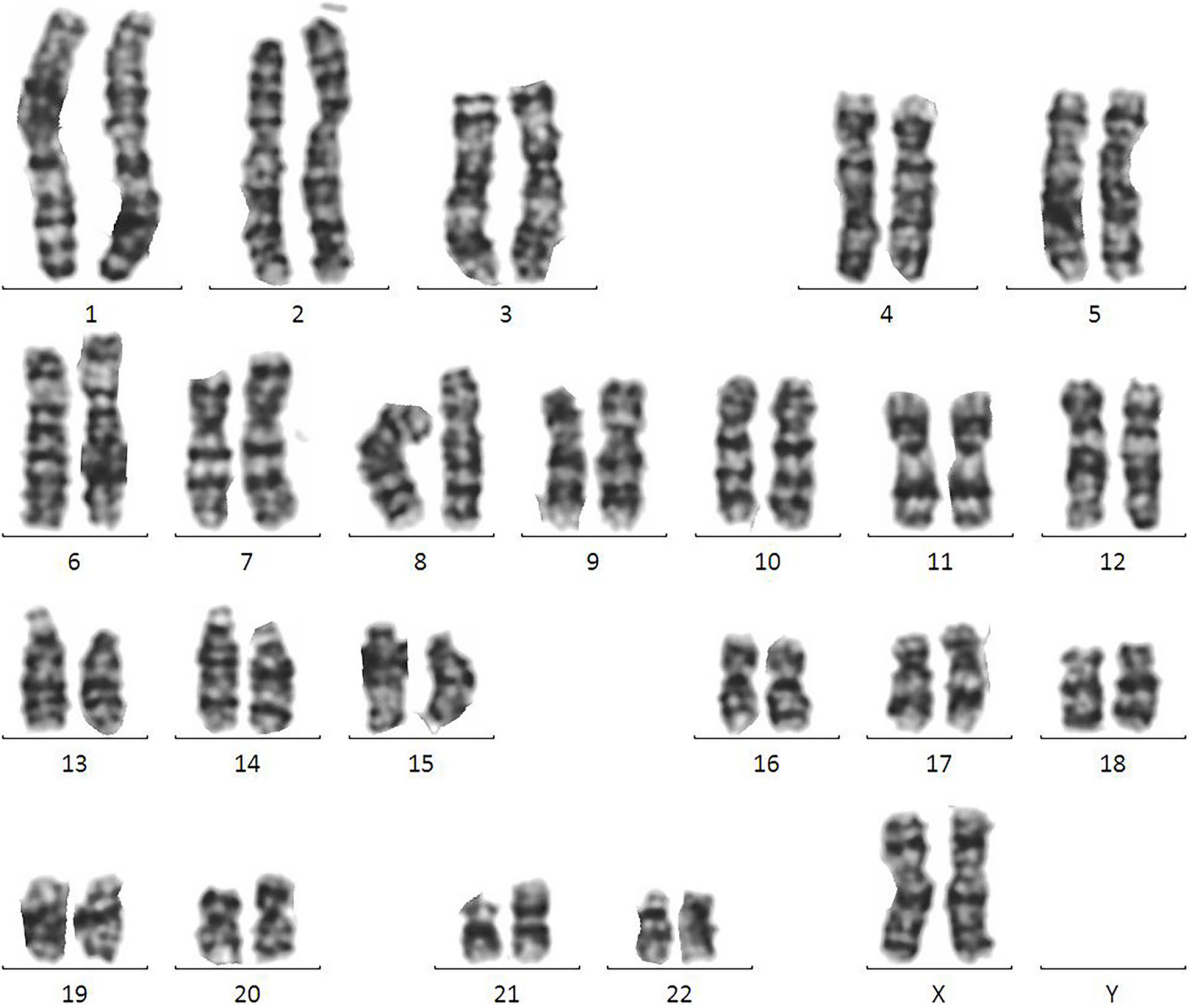
Karyotyping of the cells from twin B. A karyotype of 46, XX, + 21, der (21,21) (q10; q10) was visualized via G-banding techniques

Array-based comparative genomic hybridization (Array-CGH) analysis was performed on uncultured amniocytes with a microarray platform (Affymetrix Technologies, USA). The result confirmed the presence of three copies of chromosome 21q. And the karyotyping revealed twin B to be 46, XX, + 21, der (21;21)(q10;q10) in all 200 cultured cells (20 metaphases) (Fig. 1) karyotype of Twin A was 46, XX. Parental karyotyping from peripheral blood was normal (Mother: 46, XX; Father: 46, XY).

The couple counseled about the consequence of trisomy 21 and TTTS. They opted for selective feticide by radiofrequency ablation at 19 weeks. The procedure was uneventful. We performed a weekly ultrasound assessment for the first 2 weeks after selective fetal reduction, the DVP of the surviving co-twin became normal (30 mm) 1 week later. The surviving twin’s umbilical artery pulsatility index (UA-PI) was 1.09, and the middle cerebral artery peak systolic velocity (MCA-PSV) was 29.4 cm/s at 26^+ 4^ weeks. UA-PI was 1.43, MCA-PSV was 29.56 cm/s at 28^+ 2^ weeks. A detailed assessment of the brain, heart and limbs was performed during follow-up scans and the fetal growth was normal. Usually, the recommended time of delivery for the MCDA twin pregnancy is 36 weeks [5]. But for the management of remaining singleton pregnancy after selective feticide, we treated it as a singleton. According to Cattle’s study, after reduction to singleton pregnancies, the mean gestational age at delivery was 37.3 ± 3.7 weeks in 75 pregnancies [6]. As the survival co-twin had no fetal complications with normal growth and the mother had no pregnancy complications, she opted for spontaneous delivery under close monitoring after a detailed consultation with our obstetrician team. At 40^+ 5^ weeks gestation, twin A was born by vaginal delivery with a birth weight of 4120 g. Apgar scores were 10 and 10 at 1 min and 5 min, respectively. After birth, the baby girl’s neurologic examination was normal. She undertook regular neonatal care including regular cranial ultrasound examination, and there are not any neurological diseases such as cerebral palsy, cognitive, or motor delay. She is now 7 years old and neurodevelopmental assessment is normal.

## Discussion

Monozygotic twins are generally considered identical genetically and phenotypically because monozygotic twins arise from one fertilized oocyte. However, recent studies have reported discordance between monozygotic twins in phenotype, or genotype [7–10]. Monozygotic twins with discordant trisomy 21 are rare, with an incidence of one in 385,000 cases [11]. In the literature, all cases of monozygotic twin with discordant trisomy 21 had been reported as following karyotypes: 45,X / 46,XY; 47, XX + 21 / 46, XX; 47XY + 21 / 46XY; 47, XY,+ 21; 45, X; or 45,XY-21 (Table 2). There is no such case reporting monozygotic twins discordant for 46, XX, + 21, der (21;21) (q10; q10).

**Table 2:** Review of the cytogenetic analyses for published cases of discordant trisomy 21

study	case number	karyotype	
		twin 1	twin 2
Shapiro LR et al. 1972 [12]	2	47, XY,+21	47, XY,+ 21
		47, XY,+ 21	46 XX
Rogers JG et al. 1982 [13]	1	47,XY, +21	46 XY
Beattie RB et al. 1993 [14]	1	47,XY,+21	47,XY,+21
Nieuwint A et al. 1999 [15]	2	47,XY, +21	46,XY
		45,X/46,XY	45,X
O’Donnell CP et al. 2004 [16]	1	47,XY, +21	46,XY
Cheng P-J et al. 2006 [17]	1	45,XY,-21	46 XY
Lewi L et al. 2006 [18]	2	46,XX/45,X	46,XX
		46,XY/45,X	46,XY
Dahoun S et al. 2008 [19]	1	47,XX,+ 21	47,XX,+ 21/46,XX
Choi SA et al. 2013 [20]	1	47,XX,+ 21/46,XX	46 XX
Macatangga M et al. 2016 [21]	1	47XY + 21/46XY	47XY + 21/46XY
Chang Y-L et al. 2017 [11]	1	47, XY, + 21	46, XY

Robertsonian translocation (ROB) is a structural chromosomal rearrangement, mostly due to the fusion of two near-centromere chromosomes (human chromosomes 13, 14, 15, 21and 22) [22]. The der (21;21) (q10; q10) probably formed by a mitotic misdivision at the centromere or by an U-type exchange between sister or non-sister chromatids [23]. There are two forms of Robertsonian translocation trisomy 21: familial and de novo. On the familial form, a child’s translocation can occur by transmission from the carrier parent, while in the de novo form, parents both have a normal karyotype [24]. Since the couple’s first child is healthy and the parents’ karyotypes are normal, the current case is probably de novo.

**Fig. 2:**
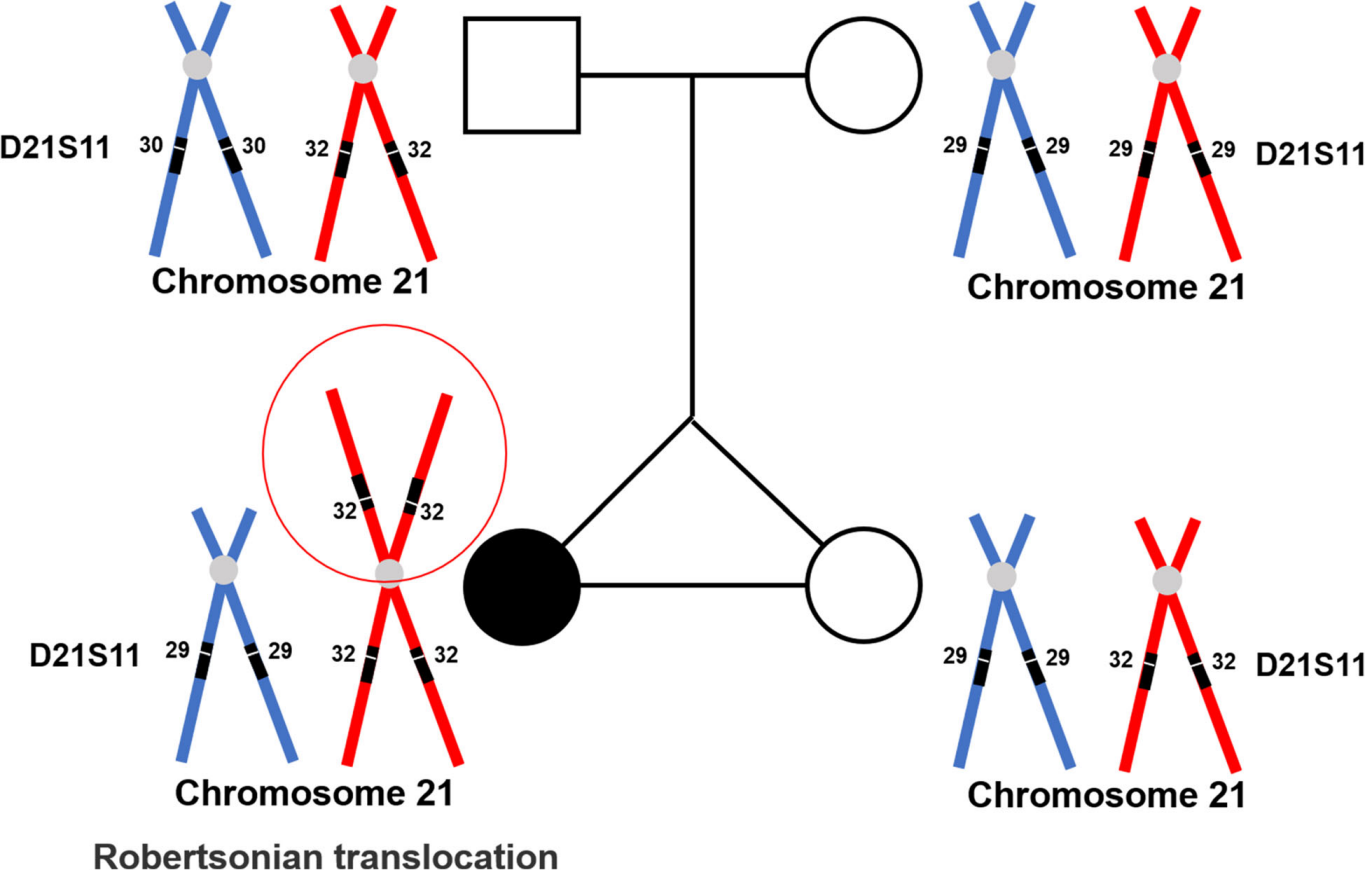
The additional chromosome 21 was the paternal origin, and uniparental isodisomy is excluded in twin A with normal karyotyping

Theoretically, two different mechanisms may lead to the molecular discovery of the current case of der (21;21) (q10;q10). Monochorionic twins with discordant trisomy 21 primarily result from prezygotic nondisjunction with trisomy rescue of chromosome 21 or postzygotic mitotic nondisjunction [25]. If ROB trisomy 21 occurred in postzygotic, usually after twinning in an initial disomic zygote, it could be caused by mitotic misdivision at the centromere or by an exchange between sister or non-sister chromatids. On the other hand, if it happened in prezygotic, paternal, or maternal meiosis might explain this event. After the zygote is formed, at the very early stage of the twinning event, one part of the inner cell mass goes through trisomy rescue to form a normal karyotype embryo, the other part proceeds to maintain the trisomy one [26]. Inefficient meiosis in both males and females may result in germ cells with missing or extra chromosomes, a chromosomal abnormality knew as aneuploidy. The aneuploidy spermatozoa level (1.8–4.5%) is lower than that of oocytes (20%). In trisomy fetuses, the additional chromosome 21 resulting from the father only accounts for 5–10% [27]. In the current case, the additional chromosome 21 was the paternal origin according to the STR markers, and uniparental isodisomy is excluded in twin A with normal karyotyping (Fig. 2).

Although laser therapy is the gold standard for stage II-IV TTTS [28], in this case, a selective fetal reduction is the right option because the abnormal fetus was a ROB trisomy 21 and associated with the cardiac abnormality. In a recent systematic review and meta-analysis, the risk of fetal loss after an amniocentesis in twin pregnancies is lower than previously reported, and there is no significant difference when comparing fetal loss before 24 weeks of gestation or within 4 weeks from the procedure in twin pregnancies undergoing with those not undergoing invasive prenatal testing [29]. This information is helpful when counseling parents about the safety of the procedure when there is indication.

## Conclusion

In conclusion, it is scarce that monozygotic twins discordant for ROB trisomy 21 of the der (21;21) (q10; q10)**,** which originates from the father and complicated by TTTS. In monozygotic twins with the discordant anomaly, the discordant karyotype should be alert.

## Supplementary Information

**Additional file 1: Supplementary Figure 1.** The discordance of nuchal translucency in this pair of twins.

**Additional file 2: Supplementary Figure 2.** The ventricular septal defect and the hypoplastic left heart in twin B.

## Data Availability

Not applicable.

## Abbreviations

MCDA: Monochorionic-diamniotic
DS: Down Syndrome
TTTs: Twin-to-Twin Transfusion Syndrome
NT: Nuchal translucency
CRL: Crown-rump length
TAPS: Twin anemia-polycythemia sequence
sFGR: Selective fetal growth restriction
VSD: Ventricular septal defect
DVP: Deepest vertical pocket
MLPA: Multiplex ligation-dependent probe amplification
STR: Short tandem repeat
Array-CGH: Array-based comparative genomic hybridization
UA-PI: Umbilical artery pulsatility index
MCA-PSV: Middle cerebral artery peak systolic velocity
ROB: Robertsonian translocation
