# Supplementary figures and images for: Monozygotic twins discordant for homologous Robertsonian translocation trisomy 21 of 46, XX, + 21, der (21;21) (q10; q10) in a twin-to-twin transfusion syndrome, case report
Source: BMC Pregnancy Childbirth. 2021 Jan 30;21:101. doi: 10.1186/s12884-021-03587-x (PMC7847594; doi:10.1186/s12884-021-03587-x)

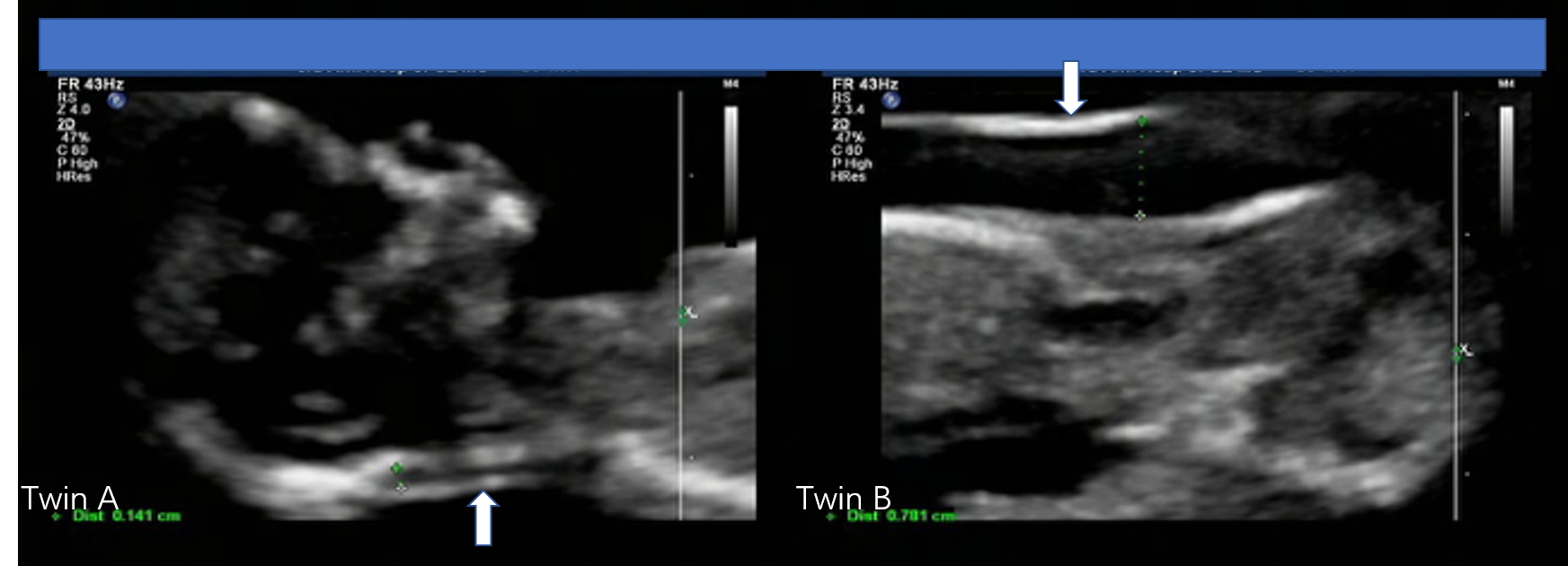

Supplement: Supplementary file 1 — Additional file 1: Supplementary Figure 1. The discordance of nuchal translucency in this pair of twins. [file 12884_2021_3587_MOESM1_ESM.tif]

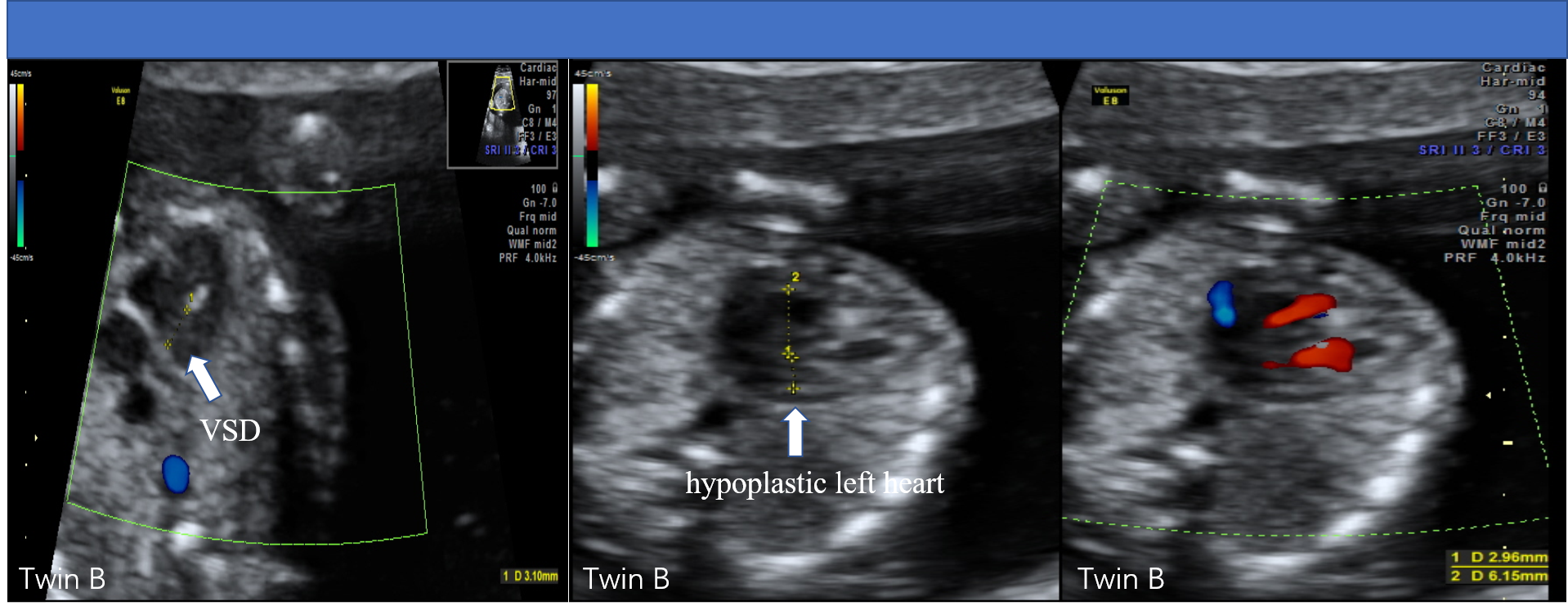

Supplement: Supplementary file 2 — Additional file 2: Supplementary Figure 2. The ventricular septal defect and the hypoplastic left heart in twin B. [file 12884_2021_3587_MOESM2_ESM.tif]
